# Pharmacokinetics of Intravenous Posaconazole in Critically Ill Patients

**DOI:** 10.1128/AAC.00242-18

**Published:** 2018-05-25

**Authors:** Fekade B. Sime, Janine Stuart, Jenie Butler, Therese Starr, Steven C. Wallis, Saurabh Pandey, Jeffrey Lipman, Jason A. Roberts

**Affiliations:** a School of Pharmacy, Centre for Translational Anti-Infective Pharmacodynamics, The University of Queensland, Brisbane, Australia; b Department of Intensive Care Medicine, Royal Brisbane and Women's Hospital, Brisbane, Australia; c University of Queensland Centre for Clinical Research, Faculty of Medicine, The University of Queensland, Brisbane, Australia; d Pharmacy Department, Royal Brisbane and Women's Hospital, Brisbane, Australia

**Keywords:** antifungal, dosing, intensive care unit, pharmacodynamics, triazole antifungal

## Abstract

To date, there is no information on the intravenous (i.v.) posaconazole pharmacokinetics for intensive care unit (ICU) patients. This prospective observational study aimed to describe the pharmacokinetics of a single dose of i.v. posaconazole in critically ill patients. Patients with no history of allergy to triazole antifungals and requiring systemic antifungal therapy were enrolled if they were aged ≥18 years, central venous access was available, they were not pregnant, and they had not received prior posaconazole or drugs interacting with posaconazole. A single dose of 300 mg posaconazole was administered over 90 min. Total plasma concentrations were measured from serial plasma samples collected over 48 h, using a validated chromatographic method. The pharmacokinetic data set was analyzed by noncompartmental methods. Eight patients (7 male) were enrolled with the following characteristics: median age, 46 years (interquartile range [IQR], 40 to 51 years); median weight, 68 kg (IQR, 65 to 82 kg); and median albumin concentration, 20 g/liter (IQR, 18 to 24 g/liter). Median (IQR) pharmacokinetic parameter estimates were as follows: observed maximum concentration during sampling period (*C*_max_), 1,702 ng/ml (1,352 to 2,141 ng/ml); area under the concentration-time curve from zero to infinity (AUC_0–∞_), 17,932 ng · h/ml (13,823 to 27,905 ng · h/ml); clearance (CL), 16.8 liters/h (11.1 to 21.7 liters/h); and volume of distribution (*V*), 529.1 liters (352.2 to 720.6 liters). The *V* and CL were greater than 2-fold and the AUC_0–∞_ was 39% of the values reported for heathy volunteers. The AUC_0–∞_ was only 52% of the steady-state AUC_0–24_ reported for hematology patients. The median of estimated average steady-state concentrations was 747 ng/ml (IQR, 576 to 1,163 ng/ml), which is within but close to the lower end of the previously recommended therapeutic range of 500 to 2,500 ng/ml. In conclusion, we observed different pharmacokinetics of i.v. posaconazole in this cohort of critically ill patients compared to those in healthy volunteers and hematology patients.

## INTRODUCTION

Treatment of fungal infections remains a significant challenge to clinicians, particularly for critically ill patients requiring management in the intensive care unit (ICU), where the incidence of invasive fungal infections and the associated mortality rate are distinctly high. In one study, for example, the overall incidence of invasive yeast infections was 16.5 cases per 1,000 admissions, and for filamentous fungi, it was 2.3 cases per 1,000 admissions (1). Although the relative incidence of invasive fungal infection is low, prophylaxis with oral/systemic antifungal agents is a very common indication due to the increasing use of immunosuppressants during critical care, cancer chemotherapy, and organ transplantation (2, 3). The success of these regimens is driven by effective dosing, with suboptimal antifungal exposures a risk factor for failure of both antifungal prophylaxis and treatment courses.

Posaconazole is an extended-spectrum triazole active against a range of yeasts and molds, including Aspergillus, Candida, *Coccidioides*, Cryptococcus neoformans, Fusarium, and *Zygomycetes* (4). It may be used for the prevention of invasive fungal infections in immunocompromised patients, including febrile neutropenic patients and those receiving immunosuppressant drugs for graft-versus-host disease during stem cell transplantation (5). It is also used for treatment of systemic fungal infections (6). The use of oral posaconazole in critically ill patients has been limited to stable patients with intact gut function, to ensure reliable bioavailability from the liquid formulation (7). Low plasma concentrations of the liquid formulation of posaconazole (mean maximum and minimum concentrations of 0.295 ± 0.152 mg/liter and 0.086 ± 0. 036 mg/liter, respectively) have been observed, in surgical ICU patients, when administered via a nasogastric tube (7). Similar findings have been reported in a general ICU population, in which a median steady-state minimum concentration of 0.167 mg/liter (interquartile range [IQR], 0.104 to 0.340 mg/liter) was observed on day 7 of therapy with a 400-mg twice-daily regimen (8). Indeed, the absence of other formulations with more reliable bioavailability has meant that oral posaconazole could not be confidently used for severe fungal infections in the critically ill. To address these concerns, a tablet formulation with enhanced bioavailability and an intravenous (i.v.) formulation were developed and are now in clinical use (9). The i.v. formulation in particular is considered a very good option in cases of gut dysfunction, which is common in critically ill patients and can lead to low posaconazole concentrations of the liquid formulation (10, 11). However, initial pharmacokinetic (PK) investigations of the i.v. formulation have been conducted only in hematology patients (9), with data in the critically ill lacking at this time.

Specific i.v. posaconazole PK data are important because critical illness is associated with pathophysiologically mediated changes in antifungal PK, which can lead to altered dosing requirements (12). Current data obtained mostly using the liquid formulation suggest that the tissue distribution of posaconazole is extensive, with a very large volume of distribution (*V*) owing to its high lipophilicity, which of itself typically means that critical illness-led PK changes may not be significant (13). Posaconazole is highly bound to plasma proteins (98 to 99%) and therefore may be affected by the presence of hypoalbuminemia, which can occur in up to 40% of critically ill patients (14). The major elimination pathway of posaconazole is through biliary excretion (about 77%) of mainly the unchanged parent compound and the rest through renal excretion as a glucuronide conjugate (13, 15). Thus, posaconazole PK are unlikely to be affected in patients with renal impairment, including those requiring renal replacement therapy (16, 17). The extent of hepatic metabolism is also limited, such that hepatic dysfunction is likely to have little effect on the need for dosing adjustment in hepatic impairment, although monitoring plasma concentration is advocated (15, 18).

Given the lack of data to guide the use of i.v. posaconazole, the objective of this study was to describe the PK of a single dose of i.v. posaconazole in critically ill patients.

## RESULTS

### Clinical data.

Eight patients were enrolled in the study. The clinical and demographic characteristics of the patients are described in Table 1. Seven of the patients were mechanically ventilated at the time of sampling, with three receiving vasopressors. One of the patients required continuous venovenous hemodiafiltration. A single dose of i.v. posaconazole was added as a second agent in the treatment of suspected yeast infection in 4 patients, while the other 4 patients had one or two yeasts identified from sterile sites, i.e., 3 patients for Candida albicans, 2 patients for Candida dubliniensis, and 1 patient each for Candida glabrata and Candida parapsilosis. Over the entire course of treatment of the above-mentioned suspected/proven fungal infections, 6 patients received fluconazole, 3 patients received voriconazole, 2 patients received caspofungin, and 1 patient received lipid complex amphotericin (Abelcet). One of the patients died with multiple organ dysfunction syndrome resulting from a perforated viscus, although this was considered not related to the study drug nor to inadequate treatment of the patient's presumed infection (no fungal pathogen was isolated).

**TABLE 1:** Clinical and demographic characteristics of patients^*a*^

Patient	Age (yrs)	Gender	Wt (kg)	BMI	APACHE II score (admission)	SOFA score		Serum creatinine (μmol/liter)	Urinary creatinine clearance (ml/min)	Serum albumin (g/liter)	ALT (IU/ml)	AST (IU/ml)	ALP (IU/ml)	Total bilirubin (μmol/liter)	INR
						Day 1	Day 2								
1	32	Female	65	25.4	17	4	2	171	34	23	57	26	124	11	1.1
2	45	Male	75	24.5	10	3	2	58	132	28	8	28	63	11	1.5
3	41	Male	46	17.1	24	3	4	84	61	16	84	126	63	8	1.2
4	36	Male	65	20.7	17	6	4	84	103	33	58	141	43	45	1.3
5	46	Male	101	31.2	17	6	1	127	59	22	28	41	84	11	1.3
6	58	Male	63.8	20.1	25	6	4	201	86	18	26	46	65	11	1.3
7	60	Male	70.4	20.3	17	2	3	43	128	18	48	47	368	8	1.4
8	49	Male	120	37.0	33	15	NA	378	0	15	66	162	102	194	2.1
Median	46		68	22.6	17	5	3	106	74	20	53	47	75	11	1.3
25th percentile	40		65	20.2	17	3	2	78	53	18	28	38	63	10	1.3
75th percentile	51		82	29.7	24	6	4	179	109	24	60	130	108	20	1.4

a Abbreviations: BMI, body mass index; APACHE II, acute physiology and chronic health evaluation II; SOFA, sequential organ failure assessment; ALT, alanine transaminase; AST, aspartate transaminase; ALP, alkaline phosphatase; INR, international normalized ratio; NA, not applicable.

### PK data.

**FIG 1:**
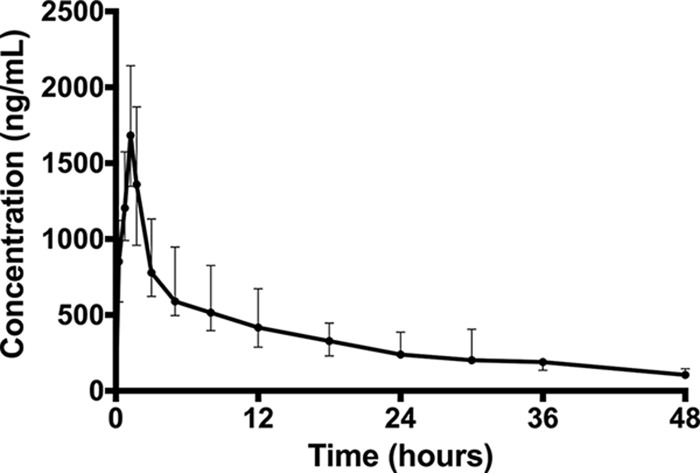
Median (IQR) posaconazole concentration versus time data from eight critically ill patients administered a single i.v. dose of 300 mg.

The plasma PK data are described in Table 2. Figure 1 shows the median (IQR) concentrations of a single dose of plasma posaconazole over a 48-h period. At 12 h, the time of usual redosing during the loading phase of i.v. posaconazole therapy, the median (IQR) concentration was 417 (288 to 672) ng/ml, and at 24 h, the median (IQR) concentration was 239 (217 to 387) ng/ml.

**TABLE 2:** Estimated posaconazole PK parameters in critically ill patients administered a single i.v. dose of 300 mg^*a*^

Patient	*C*_max_ (ng/ml)	*C*_min_ (ng/ml)	AUC_0–24_ (ng · h/ml)	AUC_0–∞_ (ng · h/ml)	AUMC_0–24_ (ng · h^2^/ml)	AUMC_0–∞_ (ng · h^2^/ml)	CL (liters/h)	λ_z_ (h^−1^)	*t*_1/2_ (h)	*V* (L)
1	1,702	92	10,202	10,505	68,898	88,077	28.6	0.066	10.5	432.7
2	1,956	250	15,129	16,996	128,426	258,915	17.7	0.046	15.2	386.5
3	2,699	668	27,692	37,323	261,386	1020,763	8.0	0.032	21.4	247.9
4	3,187	825	27,614	63,846	274,528	3283,387	4.7	0.019	36.8	249.2
5	1,703	191	12,071	18,869	114,418	733,394	15.9	0.023	29.8	684.6
6	1,351	229	11,154	14,149	99,393	331,489	21.2	0.034	20.4	625.4
7	1,353	209	8,975	12,847	75,456	398,739	23.4	0.028	24.6	828.7
8	684	220	6,814	24,766	70,537	1968,464	12.1	0.012	56.6	989.9
Median	1,702	224	11,612	17,932	106,905	566,067	16.8	0.030	23.0	529.1
25th percentile	1,352	196	9,895	13,823	74,226	313,345	11.1	0.022	19.1	352.2
75th percentile	2,141	563	18,250	27,905	161,665	1257,689	21.7	0.037	31.6	720.6

a Abbreviations: *C*_max_, observed maximum concentration during sampling period; *C*_min_, observed minimum concentration at 24 h postdose; AUC_0–24_, area under the concentration-time curve during first 24 h; AUC_0–∞_, area under the concentration-time curve from zero to infinity; AUMC_0–24_, area under the moment curve during first 24 h; AUMC_0–∞_, area under the moment curve from zero to infinity; CL, clearance; λ_z_, elimination rate constant; *t*_1/2_, elimination half-life; *V*, volume of distribution.

## DISCUSSION

To the best of our knowledge, this is the first study to describe the PK of i.v. posaconazole in critically ill patients. We observed highly variable PK parameter estimates, with wide interquartile ranges relative to the median value, in particular for the observed maximum concentration during sampling period (*C*_max_), i.e., 1,702 (IQR, 1,352 to 2,141) ng/ml, the area under the concentration-time curve from zero to 24 h (AUC_0–24_), i.e., 11,612 (IQR, 9,895 to 18,250) ng · h/ml, the AUC from zero to infinity (AUC_0–∞_), i.e., 17,932 (IQR, 13,823 to 27,905) ng · h/ml, and the volume of distribution (*V*), i.e., 529 (IQR, 352 to 721) liters or 6.7 (IQR, 5.2 to 9.4) liters/kg. The coefficients of variation for these PK parameters were 43% (*C*_max_), 55% (AUC_0–24_), 72% (AUC_0–∞_), and 49% (*V*).

The estimated *V* (median, 529 liters) is more than double the value described in healthy volunteers (236 liters) (19), suggesting that the drug is distributed more extensively into tissue. Given that posaconazole is a lipid-soluble drug, the increase in *V* could be profound in those patients with a high body weight. Indeed, the highest *V* in our cohort (989.9 liters) was observed for patient 8 (Table 1), with a body weight of 120 kg (body mass index, 37 kg/m^2^). However, previous studies with the oral formulations in healthy volunteers and hematology patients did not report a significant impact of obesity-related increase in volume of distribution on plasma concentrations (20), although the lowest *C*_max_ observed in our study (684 ng/ml) (Table 2) corresponds to the patient with the highest *V* and body weight (Table 1).

The median *C*_max_ in this study was 1,702 ng/ml, which is by far lower than the mean *C*_max_ of 2,840 ng/liter reported for healthy volunteers by Kersemaekers et al. (19). The increase in *V* (529 liters versus 236 liters) could mostly explain the lower *C*_max_ observed here, as the peak concentration is directly affected by *V* (21). However, the observed *C*_max_(1702 ng/ml) is comparable to that reported by Maertens et al. (9) in patients with hematological malignancy (1,590 ng/ml) after the first two i.v. doses of 300 mg on day 1 of treatment. That study showed that accumulation occurs after multiple i.v. doses, achieving a higher steady-state concentration (*C*_ss_) of 2,610 ng/ml on day 14.

Similar accumulation is likely in critically ill patients; however, in the current study, we have not measured steady-state concentrations to make a direct comparison, although the AUC_0–∞_ is representative of AUC_0–24_ at steady state (22). The AUC_0–∞_ in our study (after a single i.v. dose of 300 mg) was only 52% of the steady-state AUC_0–24_ (34,300 n · h/ml) reported by Maertens et al. (9) for the hematology cohort that received an i.v. dose of 300 mg twice on day 1, followed by once daily thereafter. On the other hand, the AUC_0–∞_ was only about 39% of the AUC_0–∞_ observed in healthy volunteers after a single i.v. dose of 300 mg (17,932 versus 46,400 ng · h/ml) (19). This lower exposure could possibly be due to the relatively higher rate of posaconazole clearance in critically ill patients (16.8 liters/h in this study versus 6.9 liters/h in healthy volunteers) (19). An additional factor that may have contributed to the observed low concentrations is the profound hypoalbuminemia in the studied critically ill patients. The median albumin concentration was 20 g/liter, much lower than normal values (a normal range is 35 to 55 g/liter). Because posaconazole is highly bound to plasma proteins (99%) (23), the presence of reduced albumin concentrations can affect the extent of protein binding and associated unbound plasma concentrations leading to increased distribution into tissue. This is in line with the elevated *V* we observed (Table 2). Furthermore, the increased free fraction resulting from the reduced albumin concentration means that more drug was available for elimination, consistent with the increased drug clearance observed in our patients.

The time course of exposure described by AUC_0–24_ could possibly affect the outcome of therapy, given that posaconazole is thought to exhibit both concentration- and time-dependent antimicrobial activity, which can be described by the AUC/MIC ratio (24, 25). However, pharmacodynamic studies are limited and inconsistent, and as such, the optimal dosing target is yet to be validated in a clinical study (26). Generally higher concentrations appear to increase the likelihood of improved patient outcomes (6, 10, 26, 27). Some guidelines recommend steady-state trough concentrations of >0.7 μg/ml for prophylaxis and >1 μg/ml for treatment of invasive fungal infections (3). Clearly, more data are required to accurately describe optimal plasma concentration-response relationships, as there is inconsistency between studies/authors in the recommended trough concentrations (3, 10, 15). However, it is also apparent that clinical efficacy is well correlated with plasma concentration (10). In previous studies, average steady-state concentrations (*C*_ss_) between 500 and 2,500 ng/ml, estimated by dividing the steady-state AUC_0–24_ by 24 h, has been associated with favorable outcomes in clinical trials (6, 9, 28). For the current study, the median *C*_ss_ (747 ng/ml) is within this range, and for only one of the eight patients was the *C*_ss_ less than 500 ng/ml. The maximum *C*_ss_ was 1,555 ng/ml. Therefore, the exposures achieved in the critically ill appear at the lower end of this suggested 500 to 2,500 ng/ml *C*_ss_ range, although the licensed second 300-mg dose 12 h after the first dose would mean that underdosing is unlikely in the initial phase of treatment. Furthermore, the median *C*_ss_ value (747 ng/ml) is only about half of that recently reported by Cornely et al. (1,500 ng/liter) when given as a prophylactic agent in patients with hematological malignancy (29). These observations suggest significantly different PK of intravenous posaconazole in critically ill patients with invasive fungal infections, which perhaps warrants further evaluation in a large number of patients.

On the other hand, the half-life of posaconazole in this study (median, 23 h) is comparable to that observed in healthy volunteers (mean, 24.6 h) (19). This observation is consistent with the fact that both clearance and volume of distribution were increased, which would result in a minimal change in the elimination half-life (i.e., λ_z_ = CL/*V*). The important implication of this is that the time to steady-state concentration will be unchanged in the critically ill due to the observed PK alterations, although the steady-state concentration will be lower, unless a loading dose regimen is used, as is currently recommended for i.v. posaconazole (i.e., 300 mg i.v. every 12 h for two doses and then 300 mg i.v. every 24 h). This is important, because multiple doses of intravenous posaconazole infusions are not well tolerated when administered via a peripheral catheter, with a high incidence (80%) of infusion site reactions, as previously reported (19). Such infusion-related reactions are, however, minimal with central venous catheters, as are used in critically ill patients.

This study is limited by a small sample size of only eight patients. However, this is the first study to describe the PK of i.v. posaconazole from an intensive sampling scheme over 48 h. Another limitation is that only total plasma concentrations were measured. Given the high plasma protein binding of posaconazole and the possible effect of hypoalbuminemia, further studies should aim to describe unbound posaconazole PK in plasma. Nonetheless, the interpretation of the unbound posaconazole concentration remains unclear, with limited data available relating total concentration with clinical outcomes. Lastly, the PK analysis was based on the noncompartmental approach, given that most previous population PK models described for posaconazole so far have been based on a one-compartment model (26). We recommend further studies based on the population PK approach to describe clinical descriptors of posaconazole exposure that could possibly guide future dosing.

In conclusion, the PK of a single dose of 300 mg i.v. posaconazole in critically ill patients were different from the PK data reported by Kersemaekers et al. (19) in healthy volunteers receiving the same i.v. dose of 300 mg.

## MATERIALS AND METHODS

This was a prospective, observational PK study, performed in the ICU of a 950-bed teaching hospital. The study was approved by the Royal Brisbane and Women's Hospital Human Research Ethics Committee (HREC/16/QRBW/377) and the Ethics Committee at the University of Queensland (2016001354). Informed consent was obtained from the patient or the patient's legally authorized representative.

### Patient selection.

The inclusion criteria were admission to the ICU, age of ≥18 years, the presence of suspected or confirmed fungal infection requiring systemic antifungal therapy, and the presence of central venous access for drug administration. The single dose of posaconazole was able to be given as an additional drug to other existing antifungal therapy. The exclusion criteria were pregnancy, prescription of drugs that are known to interact with posaconazole as per the product information, use of oral posaconazole within the last 2 weeks prior to enrollment, or a documented history of drug reaction to a triazole antifungal. A decision to prescribe antifungal therapy occurred as part of the routine care by the attending clinicians.

### Data collection.

Various patient clinical and demographic data, diagnoses, and microbiology data (pathogen and susceptibility, MIC, where available) were collected. Other data included the acute physiology and chronic health evaluation II (APACHE II) score on ICU admission (30), the sequential organ failure assessment [SOFA] score (31), the presence of shock and mechanical ventilation on days of sampling, renal function (serum creatinine concentrations and measured urinary creatinine clearance), hepatic function markers, concomitant medications, and the presence of renal replacement therapy (modality and settings used).

### Posaconazole administration.

In addition to the drugs given as part of the usual care as described above, a single dose of posaconazole was administered for the purposes of this study. Three hundred milligrams of i.v. posaconazole, diluted with 0.9% sodium chloride or 5% dextrose in water, was administered by slow infusion over 90 min through existing central venous access. The drug was infused through a 0.22-μm polyether sulfone (PES) or polyvinylidene difluoride (PVDF) filter.

### Sample collection.

Serial blood samples (2 ml) were collected in lithium heparin tubes immediately before and after administration of posaconazole. The first sample was collected immediately before the posaconazole infusion commenced, with subsequent samples collected during the infusion at 15 min, 45 min, 75 min, and 90 min and then at 3 h, 5 h, 8 h, 12 h, 18 h, 24 h, 30 h, 36 h, and 48 h after the commencement of drug infusion. The plasma was separated by centrifugation (3,000 rpm for 10 min) and frozen at −80°C for storage until drug assay.

### Posaconazole assay.

The total concentrations of posaconazole in plasma were measured by a validated ultrahigh-performance liquid chromatography–tandem mass spectrometry (UHPLC-MS/MS) method on a Shimadzu Nexera2 UHPLC system coupled to a Shimadzu 8030+ triple-quadrupole mass spectrometer. Plasma (10 μl) was spiked with a deuterated internal standard ([^2^H_4_]posaconazole), and proteins were precipitated with methanol. An aliquot of 2 μl of the supernatant was injected onto the UHPLC-MS/MS. The stationary phase was a C_18_ Shimadzu Shim-pack XR-ODS III 1.6-μm column (Shimadzu, Kyoto, Japan) operated at room temperature. Mobile phase A was 10 mM ammonium formate with 0.1% formic acid in water (vol/vol), and mobile phase B was 100% acetonitrile with 0.1% formic acid (vol/vol). The mobile phase of 40%A and 60%B was delivered isocratically at a flow rate of 0.4 ml/min for a 1.8-min run time and produced a back pressure of about 5,600 lb/in^2^. Posaconazole was monitored by positive-mode electrospray at multiple reaction monitoring (MRM) transitions of 701.35→127.00 (measurement) and 701.35→683.40 (reference). [^2^H_4_]posaconazole was monitored in positive mode at 705.30→127.20. The assay method was validated using the FDA criteria for bioanalysis from 20 to 5,000 ng/ml (32).

### Pharmacokinetic analysis.

The PK parameters for total posaconazole concentrations were estimated using noncompartmental methods. The area under the concentration-time curve from 0 to 24 h (AUC_0–24_) and area under the plasma concentration-time curve from time zero to infinity (AUC_0–∞_) were calculated using the trapezoidal rule. Clearance (CL) was calculated using the equation CL = dose/AUC_0–∞_. The maximum concentration for the dosing period (*C*_max_) and the minimum concentration for the dosing period (*C*_min_) were the observed values; the apparent terminal elimination rate constant (λ_z_) was determined from log-linear least-squares regression analysis of concentrations from the terminal phase; the apparent volume of distribution (*V*) equals CL/λ_z_; the half-life (*t*_1/2_) equals ln(2)/λ_z_. Figures were prepared using Prism (GraphPad, version 7.0, San Diego, CA, USA).
